# Prevalence and factors associated with of use of heated tobacco products and nicotine pouches in the European Union in 2023

**DOI:** 10.18332/tpc/218432

**Published:** 2026-06-09

**Authors:** Georgios Ktenidis, Charlotte Xin Li, Cristina Martinez, Filippos T. Filippidis, Anthony A. Laverty, Armando Peruga, Esteve Fernandez, Constantine I. Vardavas

**Affiliations:** 1 Department of HygieneEpidemiology and Medical Statistics, Medical School, National and Kapodistrian University of AthensAthensGreece; 2 Department of Primary Care and Public Health, School of Public HealthImperial College LondonLondonUnited Kingdom; 3 Tobacco Control Unit, Cancer Control and Prevention ProgramWHO Collaborating Center On Tobacco Control, Institut Català d’Oncologial’Hospitalet de LlobregatBarcelonaSpain; 4 Cancer Control and Prevention GroupInstitut d’Investigació Biomèdica de Bellvitge-IDIBELL, l’Hospitalet de LlobregatBarcelonaSpain; 5 Department of Public Health, Mental Health, and Maternal and Child Health NursingSchool of Nursing – Bellvitge Campus, Universitat de BarcelonaBarcelonaSpain; 6 CIBER en Enfermedades Respiratorias, CIBERESInstituto de Salud Carlos IIIMadridSpain; 7 Philip R. Lee Institute for Health Policy Studies, University of CaliforniaSan FransiscoUnited States; 8 Centro de Epidemiología y Políticas de SaludFacultad de Medicina, Clínica Alemana Universidad del DesarrolloSantiagoChile; 9 Secretariat of Public HealthDepartament of Health, Generalitat de CatalunyaBarcelonaSpain; 10 Department of Clinical SciencesSchool of Medicine, Bellvitge Campus, Universitat de Barcelona, L'Hospitalet de LlobregatBarcelonaSpain

**Keywords:** Eurobarometer, heated tobacco products, nicotine pouches, novel tobacco products

## Abstract

**Introduction:**

Heated tobacco products (HTPs) and nicotine pouches (NPs) are emerging nicotine containing products. This study aims to examine the prevalence and patterns of use of HTPs and NPs, and factors associated with starting to use HTPs among European Union (EU) Member State (MS) citizens.

**Methods:**

We conducted a secondary analysis of data from the Special Eurobarometer 539 survey on attitudes towards tobacco and related products, carried out between May and June 2023, which included 26359 individuals aged ≥ 15 years across the 27 EU MS. We performed two-level logistic regression models, including sociodemographic factors as independent variables, to identify factors associated with HTP and NP current and ever use and reasons to start using HTPs.

**Results:**

In 2023, 1.7% (95% CI: 1.4–1.9) of EU MS citizens were current users of HTPs and 1.0% (95% CI: 0.9–1.3) were current users of NPs; 81.2% (95% CI: 75.6–85.8) of all current HTP users were concurrently smoking tobacco, while 43.5% (95% CI: 34.4–53.1) of current NP users were also current smokers, whereas 4.3% (95% CI: 2.16–83.6) of HTP users and 18.3% (95% CI: 12.1–26.8) of NP users had never smoked tobacco. Individuals aged 15–24 years were more likely to be current users of HTPs (AOR=4.21; 95% CI: 2.67–6.64), ever users of HTPs (AOR=9.70; 95% CI: 7.66–12.29), current users of NPs (AOR=7.36; 95% CI: 4.11–13.18) and ever users of NPs (AOR=6.48; 95% CI: 4.95–8.47). Finally, individuals aged 15–24 years (AOR=5.31; 95% CI: 1.72–16.38) and 25–39 years (AOR=2.68; 95% CI: 1.19–6.42) were more likely to have reported to have commenced HTP use because they liked HTP flavors.

**Conclusions:**

Although the overall prevalence of HTP and NP use in 2023 within the EU remained low, usage was concentrated among younger individuals, with dual use with conventional tobacco products being common. The emergence of NP use among individuals who have never smoked, along with the role of HTP flavors on HTP initiation among young people, are particularly concerning.

## Introduction

Heated tobacco products (HTPs) and nicotine pouches (NPs) are emerging nicotine-containing products that have been rapidly gaining popularity globally^1,2^. Previous research in the EU had indicated that in 2020, 6.5% of the EU population had ever used HTPs and 1.3% were current users^3^. Literature suggests that younger generations are more susceptible to the use of HTPs and NPs^3-6^. However, there is limited evidence on the prevalence and factors related to nicotine pouch use in Europe. In light of the upcoming revisions of the second EU Tobacco Products Directive (TPD), which are currently underway, it is essential to assess the epidemiological trends and the characteristics of the population using these products. Thus, this study aims to examine the prevalence and patterns of use of HTPs and NPs, and factors associated with HTP initiation among European Union (EU) Member State (MS) citizens.

## Methods

### Data source and design

We conducted a secondary analysis using data from the ‘Special Eurobarometer 539 on Attitudes of Europeans towards tobacco and related products’, a cross-sectional survey implemented by the European Commission between May and June 2023 within the 27 EU MS. In brief, the survey employed a multi-stage, random probability sampling approach, selecting primary sampling points proportionally to population size and density. In total, 26359 individuals aged ≥ 15 years participated in the survey.

### Measures

#### Prevalence of heated tobacco product and nicotine pouch use

To assess the prevalence of HTPs and NPs use among EU MS citizens, we used two Eurobarometer questions^7^. For HTPs we used the question: ‘Thinking about the following product, which of the following applies to you? (Heated tobacco products)’ with possible answers being ‘You currently use it’, ‘You used to use it but you have stopped’, ‘You have tried it once or twice’, ‘You have never used it’ and ‘don’t know’. Participants who chose the first answer were categorized as current HTP users, while all those who said that they use it currently, in the past or have tried it once or twice were considered ever users. For nicotine pouches we used the question: ‘How often do you use the following products’ with possible answers being ‘Every day’, ‘Every week’, ‘Every month’, ‘Less than monthly’, ‘You used to use it regularly but you have stopped’, ‘You have tried it only once or twice’, ‘Never’ and ‘Refusal’. Individuals who replied ‘Every day’, ‘Every week’ and ‘Every month’ were grouped as current NP users, while all responses, except ‘never’ were considered as ‘ever use’.

#### Patterns of heated tobacco product and nicotine pouch use

We also examined the dual use of both HTPs and NPs and smoking tobacco. To assess the use of other tobacco products we utilized the question: ‘Regarding smoking cigarettes, cigars, cigarillos or a pipe, which of the following applies to you? (You currently smoke/you used to smoke but have stopped, you have never smoked, don’t know)’. Respondents who replied ‘you currently smoke’ were considered current users. Current users of HTPs who also reported current tobacco smoking were considered ‘dual users’ and the same was applied for current users of NPs who also reported current tobacco smoking.

#### Factors associated with decision to start using HTPs

We examined factors perceived as important in the decision to start using HTPs among current users of HTPs who do not concurrently use e-cigarettes, with the question: ‘Which of the following factors, if any, were important in your decision to start using HTPs?’. Participants could select a maximum of three answers among the following: ‘To stop or reduce tobacco consumption’, ‘They were cool or attractive’, ‘You could consume tobacco in places where tobacco smoking was not allowed’, ‘They were cheaper than other tobacco products’, ‘Your friends used HTPs’, ‘You liked the flavors of HTPs’, ‘You believed that using HTPs was less harmful than smoking cigarettes’, ‘They were more socially acceptable than smoking cigarettes’, ‘Other’, ‘None’ and ‘Don’t know’.

#### Sociodemographic data

Information were collected regarding age (15–24, 25–44, 45–64, ≥65 years), age at completion of education (≤15, 16–19, ≥20 years, still studying), gender (male, female), difficulty paying bills during the last twelve months (most of time/from time to time, almost never/never – as a proxy of socioeconomic status) and residence (rural, village, small-/middle-sized town, large town).

### Statistical analysis

We calculated the weighted proportions of individuals’ responses for the evaluated outcomes, utilizing the Stata survey-specific *svy* command and the official Eurobarometer weights. Additionally, we conducted two-level logistic regression models, accounting for clustering of observations within countries, which included the abovementioned sociodemographic characteristics as independent variables, in order to examine factors associated with current and ever use of HTPs and NPs, and reasons to start using HTPs. Results are presented as weighted percentages and adjusted odds ratios (AORs) with 95% confidence intervals (CIs). Participants who refused to reply, replied as ‘Don’t know’ to the questions related to our outcome or had missing values, were excluded from our analyses. Statistical significance was defined as p<0.05. All analyses were conducted in Stata version 19.

## Results

Our results indicate that 1.7% (95% CI: 1.4–1.9) of EU MS citizens were current users of HTPs in 2023 while 6.9% (95% CI: 6.4–7.4) were ever users. At the same time, 1.0% (95% CI: 0.9–1.3) of the EU population were current users of NPs, while 4.3% (95% CI: 3.9–4.7) had ever used NPs in 2023. As far as dual nicotine product use is concerned, 81.2% (95% CI: 75.6–85.8) of all current HTP users [1.3% (95% CI: 1.1–1.6) of EU MS citizens] were concurrently smoking cigarettes, cigars, cigarillos or pipe tobacco, while 43.5% (95% CI: 34.4–53.1) of current NP users [0.5% (95% CI: 0.3–0.6) of EU MS citizens] were also current smokers of cigarettes, cigars, cigarillos or pipe tobacco. At the same time, 4.3% (95% CI: 2.16–83.6) of HTP users had never smoked tobacco and 18.3% (95% CI: 12.1–26.8) of NP users had never smoked tobacco.

We also assessed factors associated with current and ever use of HTPs and NPs (Table 1). Younger individuals were more likely to be current and ever users, compared to those aged ≥ 55 years, with the highest AORs observed in those aged 15–24 years (current user of HTPs: AOR=4.21; 95% CI: 2.67–6.64, ever users of HTPs: AOR=9.70; 95% CI: 7.66–12.29, current users of NPs: AOR=7.36; 95% CI: 4.11–13.18, ever users of NPs: AOR=6.48; 95% CI: 4.95–8.47).

**Table 1:** Sociodemographic factors associated with current and ever use of heated tobacco products (HTPs) and nicotine pouches (NPs) in the EU, Special Eurobarometer 539, May–June 2023 (HTPs: N=25321; NPs: N=25568)

Factors	Heated tobacco products		Nicotine pouches	
	Factors associated with current use of HTPs	Factors associated with ever use of HTPs	Factors associated with current use of NPs	Factors associated with ever use of NPs
	AOR (95% CI)	AOR (95% CI)	AOR (95% CI)	AOR (95% CI)
**Gender**				
Male (ref.)	1	1	1	1
Female	1.54 (1.27–1.87)	1.09 (0.99–1.21)	0.91 (0.71–1.17)	0.69 (0.61–0.78)
**Age** (years)				
≥55 (ref.)	1	1	1	1
15–24	4.21 (2.67–6.64)	9.70 (7.66–12.29)	7.37 (4.12–13.18)	6.48 (4.95–8.47)
25–39	4.67 (3.50–6.22)	5.98 (5.15–6.94)	5.66 (3.96–8.09)	3.83 (3.24–4.52)
40–54	2.59 (1.93–3.47)	2.83 (2.44–3.29)	2.76 (1.89–4.04)	2.32 (1.96–2.75)
**Difficulty paying bills**				
Never/almost never (ref.)	1	1	1	1
From time to time/most of the time	0.96 (0.78–1.18)	0.99 (0.89–1.11)	1.08 (0.81–1.44)	1.34 (1.17–1.53)
**Age at completion of full-time education** (years)				
≤15 (ref.)	1	1	1	1
16–19	1.73 (1.07–2.79)	1.73 (1.33–2.17)	1.82 (0.97–3.39)	1.05 (0.81–1.36)
≥20	2.41 (1.47–3.93)	2.20 (1.71–2.84)	1.94 (1.02–3.66)	1.20 (0.92–1.56)
Still studying	3.31 (1.77–6.20)	2.37 (1.71–3.29)	2.23 (1.01–4.89)	1.22 (0.86–1.74)
**Residence**				
Rural (ref.)	1	1	1	1
Urban	1.48 (1.18–1.86)	1.41 (1.25–1.58)	2.14 (1.50–3.04)	1.25 (1.08–1.43)
**Smoking status**				
Never smoker (ref.)	1	1	1	1
Current smoker	46.71 (29.80–73.20)	18.29 (15.66–21.36)	8.77 (6.08–12.68)	3.92 (3.38–4.56)
Former smoker	20.13 (12.46–32.51)	9.11 (7.68–10.81)	6.05 (4.13–8.87)	3.03 (2.59–3.56)

AOR: adjusted odds ratio; from multivariate regression models and rounded to two decimal places. The models were adjusted for all factors in the table.

Finally, current users of HTPs who do not concurrently use e-cigarettes, aged 15–24 years (AOR=5.31; 95% CI: 1.72–16.38) and 25–39 years (AOR=2.68; 95% CI: 1.19–6.42) (Table 2) were more likely to state that they started using these products because they liked the flavors of HTPs, compared to individuals aged ≥ 55 years.

**Table 2:** Association of age and factors related to the initiation of using heated tobacco products among individuals who currently use or used HTPs and not e-cigarettes within the EU, Special Eurobarometer 539, May–June 2023 (N=489)

Factors	‘You liked the flavors of HTPs’	‘To stop or reduce your tobacco consumption’	‘You could consume tobacco in places where tobacco smoking was not allowed’	‘They were cheaper than other tobacco products’	‘Your friends used HTPs’	‘They were cool or attractive’	‘You believed that using HTPs was less harmful than smoking cigarettes’	‘They were more socially acceptable than smoking cigarettes’
	AOR (95% CI)	AOR (95% CI)	AOR (95% CI)	AOR (95% CI)	AOR (95% CI)	AOR (95% CI)	AOR (95% CI)	AOR (95% CI)
**Age** (years)								
≥55 (ref.)	1	1	1	1	1	1	1	1
15–24	5.31 (1.72–16.38)	0.33 (0.10–1.05)	1.02 (0.33–3.19)	3.75 (0.79–17.77)	1.33 (0.51–3.44)	1.01 (0.26–3.92)	0.7 (0.28–1.77)	2.14 (0.55–8.25)
25–39	2.68 (1.19–6.42)	0.63 (0.34–1.18)	1.1 (0.53–2.27)	4.2 (1.18–14.92)	1.07 (0.57–2.00)	1.63 (0.68–3.91)	0.46 (0.26–0.82)	1.57 (0.65–3.82)
40–54	1.57 (0.62–3.96)	0.79 (0.43–1.47)	0.38 (0.16–0.89)	1.94 (0.51–7.47)	1.46 (0.78–2.72)	1.29 (0.52–3.22)	0.56 (0.31–0.99)	1.67 (0.68–4.10)

AOR: adjusted odds ratio; from multivariate regression models and rounded to two decimal places. The models were adjusted for gender, age at completion of full-time education, difficulty paying bills, residence, and smoking status.

## Discussion

This study found that in 2023, youth were more likely to report both current or ever use of HTPs and NPs, while a substantial portion of users engaged in dual use with conventional tobacco products. At the same time, a small percentage of HTP users, but a significant portion of NP users had never smoked tobacco. Our analysis identified younger age, urban residence, higher level of education, and current or former smoking status, as significant factors associated with increased likelihood of using these novel products. In addition, among individuals who currently use HTPs, but not e-cigarettes, younger people were significantly more likely to have started using HTPs because they liked the HTP flavors.

The use of HTPs has been increasing in Europe during the last decade, with both ever and current use rising significantly^1^. Previous research highlights that younger populations and existing tobacco smokers are more likely to use these products, with dual use of conventional cigarettes reported by more than two-thirds of HTP users^6^. Regarding NPs, evidence from the US and Poland indicates higher use among men, younger individuals and current smokers, accompanied by substantial levels of dual product use^8-10^. Our results also highlight that a substantial proportion of NP users had never used smoked tobacco products. This finding raises serious concerns about the potential of the NP market’s expansion to increase nicotine exposure and addiction among non-smokers. Finally, it is evident that HTP flavors constitute a significant driver of HTP initiation among young individuals.

This is the most recent assessment of HTP use and the first cross-EU MS assessment of NP use, which comes at a crucial timepoint, as the impact of assessment of TPD2 is currently underway, with an updated TPD3 expected within the next few years^11^.

### Strengths and limitations

Our results may be subject to recall bias due to the self-reported nature of the data although self-report for cigarette smoking has been proven to be reliable^12,13^ and this is very likely to apply to new tobacco products. Also, the cross-sectional design limits our ability to infer causality or determine whether the use of HTPs and NPs led to concurrent use of conventional tobacco products, or vice versa. Eurobarometer surveys, however, employ standardized data collection methods across all EU MS, providing highly reliable and comparable data. Furthermore, the sample is representative of the EU population aged ≥ 15 years, allowing for generalization of the findings. Moreover, repeated Eurobarometer surveys using the same methodology and questionnaire^14^ will allow monitoring and characterization of these products after the revised TPD.

## Conclusions

Our study identified a higher likelihood of both HTP and NP product experimentation and use among younger populations, with dual use commonly noted. These findings indicate the need for close epidemiological surveillance of novel product use in the EU to inform regulatory decision-making.

## References

[1] Sun T, Anandan A, Lim CCW (2023). Global prevalence of heated tobacco product use, 2015-22: A systematic review and meta-analysis. Addiction.

[2] Travis N, Warner KE, Goniewicz ML (2025). The potential impact of oral nicotine pouches on public health: A scoping review. Nicotine Tob Res.

[3] Laverty AA, Vardavas CI, Filippidis FT (2021). Prevalence and reasons for use of heated tobacco products (HTP) in europe: An analysis of eurobarometer data in 28 countries. Lancet Reg Health Eur.

[4] McKelvey K, Popova L, Kim M (2018). Heated tobacco products likely appeal to adolescents and young adults. Tob Control.

[5] Felicione NJ, Ozga JE, Eversole A (2025). Oral nicotine pouches: Rising popularity and state of the science. Public Health Rep.

[6] Scala M, Dallera G, Gorini G (2025). Patterns of use of heated tobacco products: A comprehensive systematic review. J Epidemiol.

[7] European Commission (2021). Attitudes of Europeans towards tobacco and related products. https://europa.eu/eurobarometer/surveys/detail/2995.

[8] Patel M, Kierstead EC, Kreslake J, Schillo BA (2023). Patterns of oral nicotine pouch use among U.S. adolescents and young adults. Prev Med Rep.

[9] Han D-H, Harlow AF, Miech RA (2025). Nicotine pouch and e-cigarette use and co-use among US youths in 2023 and 2024. JAMA Netw Open.

[10] Jankowski M, Rees VW (2024). Awareness and use of nicotine pouches in a nationwide sample of adults in poland. Tob Induc Dis.

[11] (2014). Directive 2014/40/EU Of The European Parliament And Of The Council of 3 April 2014 on the approximation of the laws, regulations and administrative provisions of the Member States concerning the manufacture, presentation and sale of tobacco and related products and repealingDirective 2001/37/EC. Official Journal of the European Union. https://health.ec.europa.eu/system/files/2016-11/dir_201440_en_0.pdf.

[12] Patrick DL, Cheadle A, Thompson DC (1994). The validity of self-reported smoking: A review and meta-analysis. Am J Public Health.

[13] Wong SL, Shields M, Leatherdale S, Malaison E, Hammond D (2012). Assessment of validity of self-reported smoking status. Health Rep.

[14] Teshima A, Martínez C, Filippidis FT (2025). Mapping indicators of tobacco and related product use: Unveiling challenges and variations in the eurobarometer surveys over three decades. Tob Induc Dis.

